# Data to knowledge: how to get meaning from your result

**DOI:** 10.1107/S2052252514023306

**Published:** 2015-01-01

**Authors:** Helen M. Berman, Margaret J. Gabanyi, Colin R. Groom, John E. Johnson, Garib N. Murshudov, Robert A. Nicholls, Vijay Reddy, Torsten Schwede, Matthew D. Zimmerman, John Westbrook, Wladek Minor

**Affiliations:** a Center for Integrative Proteomics Research, Department of Chemistry and Chemical Biology, Rutgers, State University of New Jersey, Piscataway, NJ 08854, USA; b Cambridge Crystallographic Data Centre, 12 Union Road, Cambridge CB2 1EZ, England; c Department of Integrative Structural and Computational Biology, Scripps Research Institute, La Jolla, CA 92037, USA; d MRC Laboratory of Molecular Biology, Francis Crick Avenue, Cambridge Biomedical Campus, Cambridge CB2 0QH, England; e Biozentrum, University of Basel, Klingelbergstrasse 50-70, 4056 Basel, Switzerland; f SIB-Swiss Institute of Bioinformatics, Basel, Switzerland; g Department of Molecular Physiology and Biological Physics, University of Virginia, Charlottesville, VA 22908, USA

**Keywords:** meaning from data, big data, databases, knowledge bases, data deposition

## Abstract

Structural and functional studies require the development of sophisticated ‘Big Data’ technologies and software to increase the knowledge derived and ensure reproducibility of the data. This paper presents summaries of the Structural Biology Knowledge Base, the VIPERdb Virus Structure Database, evaluation of homology modeling by the Protein Model Portal, the *ProSMART* tool for conformation-independent structure comparison, the *LabDB* ‘super’ laboratory information management system and the Cambridge Structural Database. These techniques and technologies represent important tools for the transformation of crystallographic data into knowledge and information, in an effort to address the problem of non-reproducibility of experimental results.

## Abstract 2

This paper presents a variety of techniques and technologies aimed at the transformation of crystallographic data into information and knowledge.

## 1. Introduction   

The processing of structural information, particularly when combined with functional and evolutionary data, is a sophisticated process that requires the use of ‘big data’ paradigms for effective data management (Zimmerman *et al.*, 2014 ▶), as well as for checking data integrity and accuracy (Cooper *et al.*, 2011 ▶; Dauter *et al.*, 2014 ▶; Domagalski *et al.*, 2014 ▶; Wlodawer *et al.*, 2013 ▶). Big data traditionally refers to the analysis of very large data sets (on the scale of tera- or petabytes), and indeed the amount of data collected on a single protein crystallography synchrotron beamline station in one day may easily exceed one terabyte. However, with the steady progress in computer technology and the application of modern technologies like cloud computing, the amount of data is one of the easiest problems to deal with. The main difficulty can be summarized by the quotation, ‘Data is not information, information is not knowledge, knowledge is not understanding, understanding is not wisdom’ (attributed to Clifford Stoll and Gary Schubert; Keeler, 2006 ▶). Sophisticated technologies, including new software, must be developed to handle data management in the wet laboratory and relate it to structural and functional data. Such systems will both increase the knowledge we can derive from our data and be likely to improve data reproducibility. Similarly, new software and databases have to be developed to analyze a large number of macromolecular structures, including complexes with small-molecule agents, in the context of functional and biomedical information.

The microsymposium session ‘Data to knowledge: how to get meaning from your result’ (MS-30), held at the 23rd Congress and General Assembly of the International Union of Crystallography (IUCr; Montreal, Canada, 5–12 August 2014), was devoted to the evaluation of the current status of the road leading from crystallographic data to knowledge, and to discuss what we have to do in the future to make this road less bumpy. Several elite speakers were invited to present their work and future plans in a wide range of fields that can improve this conversion and have an impact on the issue of reproducibility of results (Carp, 2013 ▶; Collins & Tabak, 2014 ▶; Franzoni *et al.*, 2011 ▶; Prinz *et al.*, 2011 ▶). In this paper we present abbreviated summaries of these presentations.

## 2. The Structural Biology Knowledgebase: an integrated resource for all biologists   

### 2.1. Overview   

The Structural Biology Knowledgebase (SBKB, http://sbkb.org) was established to facilitate research design and analysis for a wide variety of biological systems (Gabanyi *et al.*, 2011 ▶). It serves as a single resource for a biologist, giving access to integrated sequence, structure and functional information, in addition to the available technical information reported by over two dozen contributing laboratories. The unique combination of this data allows researchers to gather new knowledge and ideas, and make informed decisions about projects in ways not possible before.

**Figure 1:**
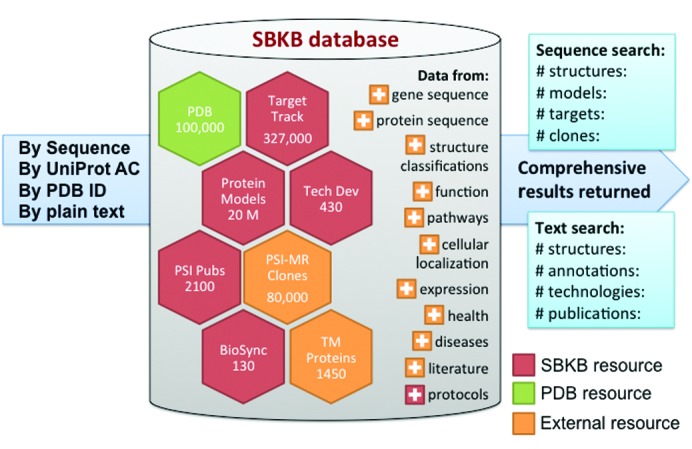
The composition of the SBKB database. The SBKB data may aggregate annotations or link to entries from 100+ public biological resources. Annotations are retrieved and reviewed for consistency weekly. A single protein or text search will pull out all instances from a wide array of data portals, curated either by the SBKB (red) or by external sources (orange), including the Protein Data Bank (PDB) archive (green).

The core SBKB database aggregates and integrates Protein Data Bank (PDB; Berman *et al.*, 2014 ▶) structures; theoretical models from the Protein Model Portal (Haas *et al.*, 2013 ▶); outcomes and experimental protocols from TargetTrack; and DNA expression clones from the PSI:Biology-Materials Repository (Seiler *et al.*, 2014 ▶), with a diverse collection of over 100 genomic, proteomic, structural, cell biological and medical data resources, encompassing functional annotations, pathways, protein expression/localization profiles, health and disease relationships, and pharmacology. As shown in Fig. 1 ▶, the SBKB can be searched by sequence, UniProt accession code or PDB ID, yielding reports combining atomic coordinates, theoretical or comparative models, annotations, experimental protocols and expression clones, ordered by sequence identity. Text searches return PDB structure hits, ranked by biological relevance (Julfayev *et al.*, 2012 ▶) or specified annotation, pertinent information from the Technology Portal (Gifford *et al.*, 2012 ▶) and relevant literature from the PSI Publications Portal. The SBKB also adds value by serving as a clarifying guide to a host of external resources. Customized ‘hubs’ were created to streamline data access for a number of important research areas (Structure–Sequence–Function resources, Homology Modeling, Transmembrane Proteins, Methods and Technologies, and Structural Targets). The SBKB also partners with Nature Publishing Group to highlight the impact of structural biology on specific areas of biological research.

**Figure 2:**
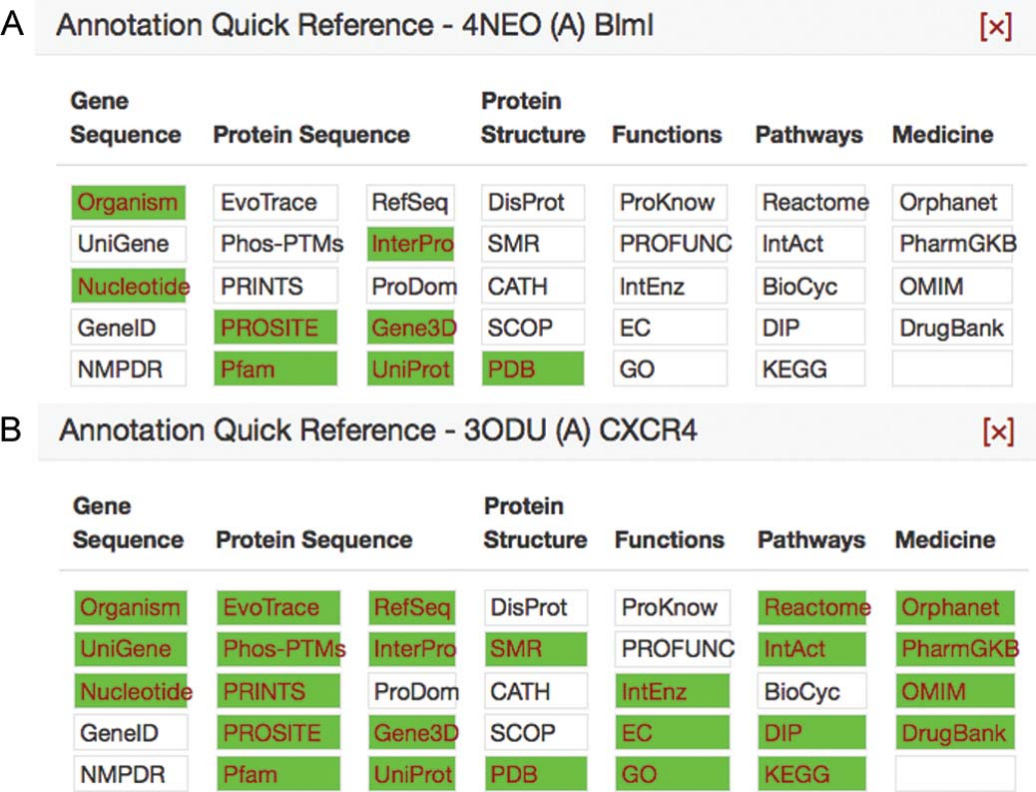
Knowledge from data known and unknown. Visual comparisons of SBKB annotation summaries give a sense of whether a protein requires further characterization, based on the number and breadth of annotations available. Knowledge of the newly studied protein *Streptomyces verticillus* BlmI (PDB code 4neo; panel A) appears more sparsely populated compared with a protein that is better understood, such as the *Homo sapiens* chemokine receptor CXCR4 (PDB code 3odu; panel B).

By aggregating this data, one can quickly view the level of knowledge there is about any given protein sequence through a single search. A user only has to know the protein’s sequence, unbiased by protein names or other text anno­tations that can change over time, to receive a list of the matching and homologous (>30% sequence identity) structures, models, target histories and DNA clones. Theoretical models, experimental progress and their annotations are still presented in the absence of an experimental structure, to provide an extended view of biology in three dimensions. The annotation notebook, which spans a wide range from gene level to medical resources, summarizes which resources have information and which ones do not, indicating directions for future research (Fig. 2 ▶).

### 2.2. Modeling and prediction tools   

Many tools have been developed by the SBKB to enable real-time predictions when ample data are not yet available. For example, the Sequence Comparison and Analysis tool (http://sbkb.org/sca) submits a construct sequence to two crystallizability prediction servers, *XtalPred* (Jahandideh *et al.*, 2014 ▶) and *Pxs* (Price *et al.*, 2009 ▶), which calculate several parameters such as isoelectric point, surface entropy and hydrophobicity, and ordered and disordered propensities. These results are combined with an SBKB report of existing structures, models and targets, so that further information about existing homologous structures, annotations, and the protocols used for both failed and successful protein production trials can be reviewed for insightful tips.

The Protein Model Portal (PMP), which houses over 20 million pre-computed comparative models of protein sequences, has also created a real-time interactive modeling tool that will submit the user’s sequence to eight partner servers for possible novel modelling or re-modeling based on newer structural templates. Since model quality determines the usability of a model for specific applications, the PMP helps assess the reliability of the new models by submitting it to three well established quality estimation servers. Since there are >50 000 000 sequences in UniProtKB, and now >100 000 structures in the PDB, such models can be very useful for hypothesis-driven research in the absence of an experimental structure.

Additional search tools have been developed to predict and locate structures based on their function. The KB-Rank tool (http://protein.tcmedc.org/KB-Rank/) is a text search tool that retrieves a list of protein structural chains related to functional or disease-related annotations (Julfayev *et al.*, 2012 ▶). Its unique feature is that structural chains within each retrieved category are ranked according to their estimated relevance to the queried text, based on their prevalence (frequency) in the literature and in annotation resources. The KB-Role tool (http://protein.tcmedc.org/KB-Role/) uses information derived from a protein sequence and three-dimensional structure to predict a likely Gene Ontology term association (Julfayev *et al.*, 2011 ▶). Each prediction has an assigned probability value, so a user can assess whether it is to be considered for further study.

### 2.3. Quality assurance   

The SBKB also strives to deliver only high-quality curated data from established resources, and its developers are keenly aware of the dangers of cyclical propagation of incorrect annotations. To power our search tools, we perform a weekly review of the collected annotations for errors and inconsistencies, and resolve them with the provider. Over the years, we have worked with model organism databases and EBI annotation teams, resolving issues ranging from improper mappings of structural annotations in non-structurally determined regions, to corrupted output files resulting in lack of data, to changes in data delivery format and nomenclature. Such audits are required to ensure that the community always receives the full and latest compliment of annotations available, from SBKB and other resources.

In this era of big data, there is still much to be done to align all of the data housed with public biological databases so that further biological knowledge can be realised. The SBKB represents the first step towards making that a practical reality.

## 3. Virus maturation and the VIPERdb virus structure database   

### 3.1. Overview of VIPERdb   

The Virus Particle ExploreR (VIPERdb) database provides the non-expert in structural virology with access to the coordinates from the 420 X-ray crystallography structures determined for viruses with icosahedral symmetry (Carrillo-Tripp *et al.*, 2009 ▶). The size range of these particles extends from 150 Å, for the smallest viruses formed by 60 copies of the same gene product, to over 1000 Å, for adenovirus formed by 13 different gene products (Benevento *et al.*, 2014 ▶; Reddy & Nemerow, 2014 ▶). Each virus has a main page that provides details of both the virus and the structural study, and this can be found by virus name, PDB code, or as a member of a subset based on the family name or *T* number. The coordinates for all the viruses are organized relative to the same orthogonal coordinate system, allowing straightforward comparisons and operations among the entire database. A variety of options are available for displaying the virus particle, including rendered volumes color-coded by subunit type, color-coded by radius, displayed with a ‘cage’ that shows the quasi-equivalence of the capsid, or a ribbon drawing of the subunits in the icosahedral asymmetric unit. Based on their standard labeling, the coordinates of any oligomer of icosahedral asymmetric units can be downloaded for analysis and display using a graphics program of the user’s choice. There are a variety of derived results available directly as tables or graphs, such as the buried surface area at the unique subunit interfaces and the amount contributed by each residue at the interface. Stabilization energy is estimated from the buried surface, giving the contribution of each residue to the overall stability of the interface. Comparisons of derived results can be made among members within a virus family through a graphical user interface (GUI) that directly provides all of these for each virus, and these can be sorted on any given property. Visual comparisons among all the viruses in the database can be made through the gallery maker. Viruses for comparison can easily be selected and displayed on the same relative scale in a single image. The best way to become acquainted with the database is to begin using it. There is an extensive tutorial provided, but most of the operations are intuitive and accessible to the non-expert with little training.

### 3.2. A case study: maturation of *Nudaurelia Omega Capensis* virus   

**Figure 3:**
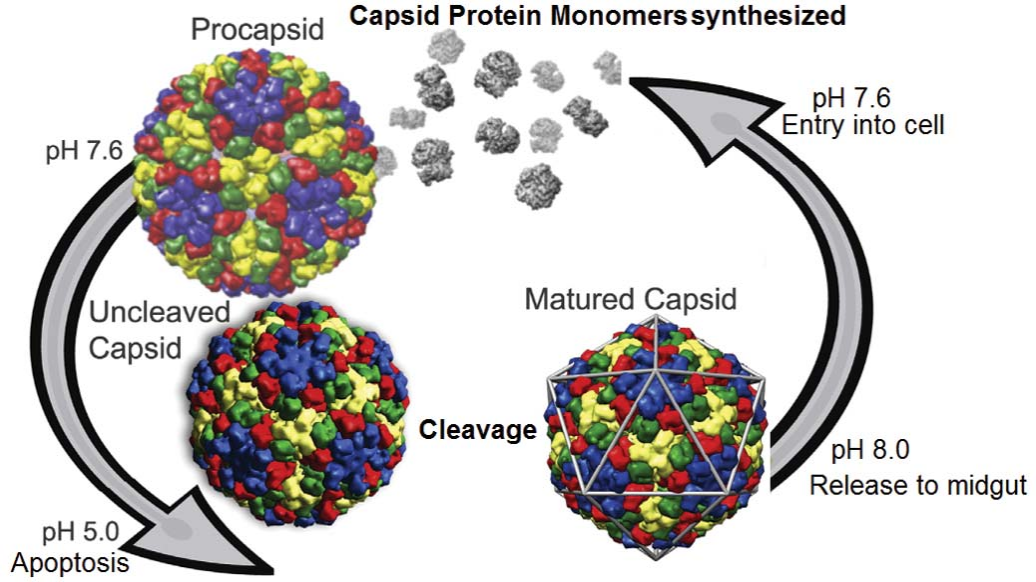
The life cycle of tetraviruses. Infected cells produce procapsids at neutral pH. Virus infection eventually triggers apoptosis, which induces a drop in pH and virus maturation. Release to the alkaline mid-gut allows the virus to infect new cells and start the cycle again.

A study of virus maturation provides an example of the use of VIPERdb. *Nudaurelia Omega Capensis* virus (NWV) is a non-enveloped single-stranded RNA insect virus with *T* = 4 quasi-symmetry, *i.e.* the particle contains four copies of a single type of gene product (644 amino acids) in the icosahedral asymmetric unit, creating local two-, three- and sixfold quasi-symmetry axes in addition to the icosahedral symmetry (Dorrington & Short, 2010 ▶). NWV undergoes large-scale particle reorganization between the immature procapsid and the mature capsid, as well as an autocatalytic cleavage of the subunits between residues 570 and 571 (Canady *et al.*, 2000 ▶) (see Fig. 3 ▶). The virus initially assembles at neutral pH within the gut cells of *Lepidoptera* larvae and matures late in the infected cell when the cell undergoes infection-induced apoptosis, with an associated reduction in pH to 5 (Tomasicchio *et al.*, 2007 ▶). The process can be recapitulated *in vitro* by expressing the capsid protein of NWV in a baculovirus system and purifying the procapsids. Maturation does not take place in the baculovirus system because the SF21 cells used for expression do not undergo apoptosis.

VIPERdb was used to analyze the residues at subunit interfaces determined by the 2.8 Å crystallographic analysis of the mature virus (Helgstrand *et al.*, 2004 ▶). As expected for pH-dependent structural changes, a large number of acidic residues were found at the subunit interfaces. The X-ray coordinates were used for computing the electrostatic potential of the subunit surfaces at pH 7.5 and 5.0, demonstrating the large change in electrostatic repulsion between the two pH values (Matsui *et al.*, 2009 ▶). Purified procapsids were titrated at pH intervals of 0.2 between 7.6 and 5.0, and their size distribution examined by small-angle X-ray scattering (SAXS) (Matsui, Tsuruta & Johnson, 2010 ▶). The particle population was essentially uniform at each pH value, as demonstrated by the precise fitting of spherical models to the SAXS data. The resulting titration curve based on radius showed that the overall p*K_a_* of the particle is 5.9. Maturation cleavage initiated at pH 5.5, but would not go to completion within 24 h unless the pH was lowered to 5.2. The kinetics of the cleavage were measured by the change in Coomassie stain in sodium dodecyl sulfate–polyacrylamide gel electrophoresis (SDS–PAGE) patterns at the mass corresponding to full length (644 amino acids) and at the mass of the cleaved subunit (570 amino acids). At pH 5, half of the subunits cleaved in 30 min, but it took another 4 h for the remaining subunits to cleave. This suggests that the four subunits in the icosahedral asymmetric unit cleave at different rates depending on their quaternary structure position. Subunits designated *A* form pentamers, while subunits labeled *B*, *C* and *D* form quasi-hexamers at the icosahedral twofold symmetry axes. *D* subunits cluster about the icosahedral threefold symmetry axes, while *A*, *B* and *C* form a similar quasi-threefold axis. The *DDD* and *ABC* trimers are related by a quasi-twofold axis.

Time-resolved electron cryomicroscopy (cryoEM) and image reconstruction were performed by flash-freezing samples at 3 min, 30 min and 4 h after lowering the pH from 7.6 to 5.0 and computing sub-nanometre reconstructions. An additional sample was incubated for two weeks at pH 5.0 (fully mature) and a sub-nanometre reconstruction computed. Difference maps were computed between the reconstructions at each time point and for the fully mature particle at grid points surrounding the cleavage sites (known from the X-ray model). Assuming that large differences corresponded to subunits that had not cleaved and small differences to those that had cleaved, it was clear that the *A* and *D* subunits cleaved first, *B* was slower and *C* was the slowest (Matsui *et al.*, 2010 ▶). Employing the same data, but in an entirely different way, it was shown that regions around the cleavage site for the *A* and *D* subunits had the least variance, while the same regions had the greatest variance for *B* and *C* over the ensemble of particles at the 3 and 30 min time points, implying that the cleavage site had formed for *A* and *D* and was still in the process of forming for *B* and *C* (Wang *et al.*, 2013 ▶).

Two roles were demonstrated for the cleavage. First, maturation is not reversible in wild-type NWV (Canady *et al.*, 2001 ▶). However, mutating Asn570 to Thr and Glu103 to Gln inhibits cleavage and the maturation reorganization is reversible when the pH is raised from 5 to 7.6 (Taylor *et al.*, 2002 ▶). It was shown that cleavage allows residues 571–644 of the *D* subunit to form a molecular chock properly, while these residues are disordered at pH 5 when cleavage has not occurred (Tang *et al.*, 2009 ▶, 2014 ▶). Secondly, it was shown that cleavage is required for particle interactions with liposomes and the associated formation of pores in artificial membranes (Domitrovic *et al.*, 2012 ▶). Such lytic activity has been found in all non-enveloped viruses studied and is associated with a ‘fusion-like’ peptide essential for infectivity (Banerjee & Johnson, 2008 ▶). While residues 571–644 in *D* subunits have a clear structural role in stabilizing the particle, some of the same residues in *A* subunits form a helical bundle (571–595 helical and 596–644 invisible) at the pentamer axes and are poised for release with the correct environmental cue (Helgstrand *et al.*, 2004 ▶; Domitrovic *et al.*, 2012 ▶). Rapid cleaving of *A* and *D* provides early structural stability and the lytic activity essential for infectivity.

### 3.3. Future directions   

Maturation of NWV provides an excellent opportunity to follow a large-scale reorganization of a virus particle in a frame-by-frame manner by carefully controlling the pH and doing high-resolution cryoEM reconstructions at the pH intervals. The availability of direct electron detectors makes it likely that intermediate structures can be determined at 4 Å resolution or better.

## 4. Expanding our knowledge of the protein universe: modeling protein structures by homology   

### 4.1. Protein structure homology modeling   

Computational modeling and prediction of three-dimensional macromolecular structures and complexes from their sequence has been a long-standing goal in computational structural biology. As a result of the data deluge generated by large-scale sequencing efforts, the number of amino-acid sequences in public databases such as UniProt (UniProt Consortium, 2014 ▶) has been rising exponentially, outgrowing the number of experimental structures deposited in the PDB at the same time by orders of magnitude. Fortunately, most of the increase in complexity observed in new sequencing data is not due to the discovery of new protein domain families, but to permutations of domains which have previously been observed in other proteins (Levitt, 2009 ▶). Therefore, computational approaches for modeling proteins using comparative methods (homology modeling) have become an important tool for extrapolating the available experimental structure information to new protein sequences without direct structure information (Baker & Sali, 2001 ▶). Methods for structure modeling and prediction have made substantial progress over the last few decades, and template-based homology modeling techniques have matured to stable and reliable pipelines which are now routinely used to complement experimental techniques. More than 20 years ago, *SWISS-MODEL* pioneered the field by providing the first fully automated structure modeling service on the internet (Biasini *et al.*, 2014 ▶; Guex *et al.*, 2009 ▶; Peitsch, 1995 ▶). Today, a broad variety of structure modeling services are available publicly (Hildebrand *et al.*, 2009 ▶; Pieper *et al.*, 2014 ▶; McGuffin & Roche, 2011 ▶; Raman *et al.*, 2009 ▶; Zhang, 2014 ▶). The Protein Model Portal (Arnold *et al.*, 2009 ▶) of the SBKB (Gabanyi *et al.*, 2011 ▶) aims to offer a ‘one-stop shop’ for structure information, both models and experimental structures.

Over the last two decades, we have observed a paradigm shift in structural biology, starting from a situation where a large ‘knowledge gap’ between a huge number of protein sequences contrasted with a relatively small number of experimentally known structures often impeded the systematic use of structural information in biomedical research (Baker & Sali, 2001 ▶; Schwede *et al.*, 2000 ▶). Over the last few years, experimental structures have been solved for a significant fraction of all protein families, and today some form of structural information – either experimental or computational – is available for the majority of amino acids encoded by common model organism proteomes (Schwede, 2013 ▶). Not surprisingly, computational structure models are used routine­ly in a broad spectrum of biomedical applications (Schwede *et al.*, 2009 ▶).

### 4.2. Assessment of homology modeling methods: CASP and CAMEO   

Unfortunately, computational modeling and prediction techniques often fall short in accuracy compared with high-resolution experimental structures, and it is often difficult to convey the expected accuracy and structural variability of a specific model. Retrospectively assessing the outcome of blind structure predictions in comparison with experimental reference structures allows one to benchmark the state-of-the-art and identify areas which need further development. The critical assessment of structure prediction (CASP) experiment has, for the last 20 years, assessed progress in the field of protein structure modeling based on predictions for *ca* 100 blind prediction targets per experiment, which are carefully evaluated by human experts (Moult *et al.*, 2014 ▶; Moult, 2005 ▶). The continuous model evaluation (CAMEO) project (Haas *et al.*, 2013 ▶) aims to provide a fully automated blind assessment for prediction servers, based on weekly pre-released sequences of the PDB. CAMEO has been made possible by the development of novel scoring methods, such as the local distance difference test lDDT (Mariani *et al.*, 2013 ▶) or CAD score (Olechnovič *et al.*, 2013 ▶), which are robust against domain movements and allow for automated continuous structure comparison without human intervention.

One important outcome of these analyses is that the quality differences observed between methods are negligible compared with the differences in accuracy between easy and hard prediction targets (Huang *et al.*, 2014 ▶; Mariani *et al.*, 2011 ▶). Reliable estimates of the quality for individual models are therefore crucial to define the range of applications for which a specific model is likely to be suitable (Schwede *et al.*, 2009 ▶). Validation methods which can estimate the local quality of models on an absolute scale are required, and various approaches have been developed by the modeling community. Their performance can be evaluated independently by the mechanisms of CASP (Kryshtafovych *et al.*, 2014 ▶) and CAMEO (Haas *et al.*, 2013 ▶). While single-model methods, *e.g.* based on statistical potentials, are able to assess individual models (Ray *et al.*, 2012 ▶; Benkert *et al.*, 2011 ▶; Wiederstein & Sippl, 2007 ▶), they are in general not as accurate as consensus-based approaches (Skwark & Elofsson, 2013 ▶). However, these methods require an ensemble of independent models to be provided. Quasi single-model methods overcome this limitation by creating a model ensemble ‘on the fly’ so that, from a user perspective, the assessment of a single model becomes possible (Roche *et al.*, 2014 ▶).

### 4.3. Future directions   

While comparative modeling methods have made substantial progress over the few last decades, significant challenges still exist and these are the target of active research in the modeling community, such as modeling oligomeric states and complexes (Biasini *et al.*, 2014 ▶; Shapovalov *et al.*, 2014 ▶), modeling the binding sites of functionally relevant ligands and cofactors (Gallo Cassarino *et al.*, 2014 ▶), refining models closer to the native structure (Nugent *et al.*, 2014 ▶) or predicting the substrate specificity of enzymes (Tian *et al.*, 2013 ▶).

## 5. Conformation-independent structural comparison of macromolecules with *ProSMART*   

Comparative structural analyses are often performed in order to identify particular residues or regions that may be important for global or local fold stability or biological function, allowing the investigation of potential functional relationships and evolutionary links. The identification and exploration of (dis)similarities between macromolecular structures can help to provide biological insight, for instance when visualizing or quantifying a protein’s response to ligand binding. Obtaining a residue alignment between compared structures is generally a prerequisite for such comparative analysis.

There have been various approaches developed for the alignment and comparison of macromolecules, some of which require global spatial rigidity, whereas others permit more flexibility, allowing alignment in the presence of domain motion [see *e.g.* Krissinel (2012 ▶) or Ye & Godzik (2003 ▶), to name but two; for a more detailed overview, see Nicholls (2011 ▶)]. Traditionally, the structural alignment problem has often been considered analogous to that of fold recognition, which exacerbates the commonly perceived ambiguity between the terms ‘alignment’ and ‘superposition’. However, if the conformational difference between the compared structures is dramatic or complex, conventional alignment methods may struggle to provide an intuitive solution for straightforward analysis.

Indeed, it can often be hard to identify or quantify subtle differences between models, especially when attempting to do so by simply superposing structures and inspecting them manually. This can be even more challenging when the compared models cannot be easily or unambiguously superposed, such as when the models undergo conformational change, which may be due to effects that are biologically relevant such as binding, or due to environmental factors such as crystal packing. However, this task can be made dramatically easier by investigating the conservation of local structure, which can provide great insight. Whilst there are many alignment tools that optimize a superposition, there has been a need for methods that compare macromolecular structures in a way that is independent of the global conformations of the compared models.

There are often distinct measurable structural differences between highly homologous crystallographically determined macromolecular models. Such differences may occur at both global and local levels, and may be due to biologically relevant factors or to the influences of crystal content and/or packing. Equally, it is often of relevance to analyze the structural variability of model ensembles achieved using other experimental or theoretical methods, such as electron microscopy, NMR spectroscopy and molecular dynamics simulations. At the global level, structural differences include domain motion (*e.g.* due to molecular binding), domain distortion (*e.g.* due to crystal packing) and more dramatic conformational changes (*e.g.* domain swaps, alternative folds). At the local level, differences include changes in backbone and side-chain conformation, which may be subtle or dramatic, and which may or may not be of particular biological interest. Generally, identifying both regions that are and those that are not locally conserved can provide useful information during a comparative analysis. Such information cannot be easily inferred using a simple superposition, and thus is often masked when using traditional representations. As such, the development of techniques dedicated to this task has been required, and this demand motivated the development of *ProSMART*.

### 5.1. *ProSMART* structural comparison   

The conformation-independent structural comparison tool *ProSMART* (Procrustes Structural Matching Alignment and Restraints Tool) is designed to allow fast but detailed comparative analysis of macromolecular structures in the presence of conformational changes. *ProSMART* is suited to the analysis of the structural conservation of the local backbone and side chains in a wide variety of scenarios. The approach is sensitive enough to allow the identification of subtle dissimilarities between structures sharing a high sequence homology, whilst being versatile enough to scale to the identification of surprising local similarities between more distantly related structures.

*ProSMART* aligns contiguous backbone fragments using a dynamic programming algorithm, and subsequently compares the matched structures in order to analyze local structural conservation of the compared macromolecular models (for details, see Nicholls *et al.*, 2014 ▶). Being primarily interested in the conservation of local backbone structure, the initial alignment stage is completely independent of spatial relationships. However, following alignment, the spatial relationships of matched backbone fragments are analyzed in order to identify the presence of rigid substructures. Specifically, the conformation-independent fragment alignment is utilized in identifying clusters of aligned fragment pairs that belong to the same coordinate frame (for details, see Nicholls, 2011 ▶). Such clusters may correspond to rigid structural units, *e.g.* domains, and are used to superpose separately each identified shared substructure. Subsequently, the angular differences between the substructures are identified, allowing differences in global conformation (*e.g.* due to domain motion) to be described using an axis-angle representation. This method greatly contrasts with conventional r.m.s.d.-based approaches; the resulting superposition is not based on the whole domain, but rather on the notion of the substructure’s average co­ordinate frame, allowing a tighter superposition of the substructure’s core.

*ProSMART* allows structural comparisons to be performed at a chosen level of structural resolution (note that this does not refer to crystallographic resolution, but rather to the level of structural detail), since the backbone fragment length may be selected as desired. Performing the analysis at varying levels of structural resolution can provide useful and complementary insight regarding conformational differences between the compared models, allowing the extraction of a rich breadth of information that may be used to examine the nature of any observed (dis)similarities more closely. For example, choosing a short fragment length (three to five residues) results in performing analyses at a high level of structural resolution, which could be useful for the highly sensitive analysis of local backbone curvature in hinge regions. In contrast, choosing a long fragment length (more than nine residues) would operate closer to the secondary structure level, smoothing out any finer details and providing a more stable lower-resolution view, whilst being more affected by larger conformational differences between the compared structures. A default analysis would typically be performed using intermediate fragment lengths (seven to nine residues), offering a reasonable trade off between sensitivity, stability and conformation independence.

**Figure 4:**
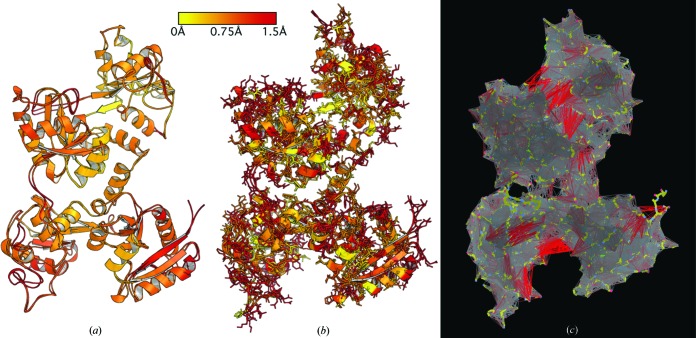
*ProSMART* structural comparison of macromolecules during crystallographic refinement. Comparative analysis of the 3.5 Å model 1ryx of ovotransferrin, before and after re-refinement with external restraints from the sequence-identical 2.15 Å model 2d3i, which adopts a different global conformation. For clarity, the reference model 2d3i is not shown. Details of the re-refinement of 1ryx using 2d3i as a reference structure are detailed elsewhere (Nicholls *et al.*, 2013 ▶). The models are superposed and colored according to (*a*) local backbone dissimilarity and (*b*) side-chain dissimilarity using a color gradient (yellow implies similarity, red relative dissimilarity), displayed using *PyMOL*. These representations allow a quick visual identification of which regions of the backbone and side chains have dramatically changed conformation during refinement. In this case, it is evident that there were substantial changes to the local structure but no changes to the global conformation during refinement. (*c*) Using *Coot* (Emsley *et al.*, 2010 ▶) to visualize the external restraints used during refinement provides information regarding the nature of the external restraints, which are represented as interatomic lines colored gray to red, indicating the similarity of the restraint target values to the current interatomic distances. The prevalence of restraints colored red between domains is due to differences in global conformation between the target and reference models; these restraints would have little effect during refinement due to being down-weighted by *REFMAC5*.

The comparative analysis features of *ProSMART* can be useful in a wide variety of scenarios, providing the ability to analyze structures at varying levels of detail. For example, near-identical models may be compared at a very high level of detail, investigating subtle differences between corresponding backbone regions or side chains. This could be used to investigate the influence of different environmental conditions (*e.g.* different ligand binding modes, different crystal contacts *etc.*) or to assess the extent of the change a model undergoes during the crystallographic model building and refinement process (see Fig. 4 ▶). Comparative structural analysis at more moderate levels of detail may be performed on highly homologous structures, often those which adopt slightly or substantially different global conformational states. The evaluation of such conformational changes may involve the identification of residues of interest, a description of any hinging motions and an assessment of internal surface loop variability. At a lower level of detail, the backbone scores provided by *ProSMART* are able to distinguish between varying levels of local dissimilarity, irrespective of the overall similarity between the compared structures. In practice, this can be useful for the identification of local similarities between seemingly dissimilar structures and the visualization of local dissimilarities in corresponding regions of homologous structures, noting that chains exhibiting the same global fold but no conservation of local structure cannot be meaningfully compared in this way (other than to clarify that local structure is not conserved). In addition, *ProSMART* can be used to assess the degree of local structural dissimilarity over multiple homologous models.

### 5.2. Presentation of results   

*ProSMART* reports various residue-based local dissimilarity scores pertaining to the conservation of backbone and side-chain conformation, which can be used in concert to analyze the local structural environments of the residues (see Fig. 4 ▶). Scores that relate to the raw structural dissimilarity of the residues’ immediate local backbone environments help to identify whether structural regions are internally near-identical, irrespective of whether or not the compared models adopt dramatically different global conformations. The degree of rotational hinging of the backbone about each residue is also reported; this measure is highly sensitive to any backbone curvature or torsion, allowing the identification of any regions that exhibit subtle backbone deformation. In addition, *ProSMART* provides measures of the structural conservation of side chains relative to their local coordinate frames. This functionality may be used to compare close homologues, whether in the same or different global conformational states, allowing the immediate location of side chains that adopt similar or different conformations in the compared models. This can be useful in various situations, *e.g.* if the user wishes to investigate and visualize differences in side-chain conformation at sites of interest, or study the effects of external influences such as small-molecule and metal binding, bio­logical assembly and crystal packing.

The provision of various residue-based local dissimilarity scores for the backbone and side chains, and the ability to view results intuitively in color using the molecular graphics software *CCP4mg* (McNicholas *et al.*, 2011 ▶) and *PyMOL* (Schrödinger, 2010 ▶), provides a unique and informative way of performing comparative structural analyses. Residues are colored using an intuitive gradient (colors and gradient scales may be chosen) representing various levels of dissimilarity. This default output can provide useful information that may be hard to achieve manually, and at the same time easily produce quality graphical representations of structural analyses. In particular, the *ProSMART* interface within *CCP4mg* offers useful functionalities, including the ability to alter colors and gradients in real time. *ProSMART* is available as a stand-alone package, as well as being distributed as part of the *CCP4* suite (Winn *et al.*, 2011 ▶), and can currently be executed either as a command-line tool, through the *CCP4i* GUI (Potterton *et al.*, 2003 ▶) or *via* *CCP4mg*.

### 5.3. Application of *ProSMART* in macromolecular crystallographic refinement   

In addition to being used for comparative structural analysis, *ProSMART* is also used for the generation of external interatomic distance restraints for use in low-resolution macromolecular crystallographic refinement by *REFMAC5* (Murshudov *et al.*, 2011 ▶) and in real-space refinement by *Coot* (Emsley *et al.*, 2010 ▶). The adopted alignment approach is considered appropriate for this application since the generated restraints operate locally, being independent of global conformational differences between the target and reference structures (Nicholls *et al.*, 2012 ▶). The structural comparison and restraint generation features of *ProSMART* can also be used to aid the refinement of macromolecular models into cryoEM maps (Brown *et al.*, 2015 ▶).

Regularizers are used to stabilize macromolecular crystallographic refinement and to ensure consistency between the derived models and available prior knowledge. At low resolution, a weak signal, noisy data and a poor observation-to-parameter ratio often cause unstable refinement with a higher risk of over-fitting, and ultimately result in an unreliable model. Such complications during refinement can be lessened by the introduction of additional regularizers such as external restraints. These restraints are designed to utilize structural information as a source of prior knowledge, helping local interatomic distances to agree with previous observation without inappropriately enforcing global rigidity. Such structural information may be derived from homologous models where available, even if in a different global conformational state or from a different crystal form. Otherwise, more generic types of information can be utilized, such as knowledge of hydrogen-bonding patterns or the typical conformations of secondary-structure elements and other structural fragments. External restraints generated by *ProSMART* are typically short (2.5–4.2 Å), stabilizing local structure whilst allowing global conformational flexibility between target and reference structures. External restraints output by *ProSMART* can be visualized, analyzed and edited in *Coot* (see Fig. 4 ▶).

Challenges when using external restraints include the determination of suitable reference structures and ensuring robustness to inappropriate restraints. The structural analysis features of *ProSMART* are intended to aid such assessment, allowing quantitative and visual analysis of localized differences between related structures. These features are useful for comparing target and reference structures, and for investigating the extent of any local backbone and side-chain structural changes that may occur during the model building and refinement process. Indeed, the comparative structural analysis features of *ProSMART* can be useful during crystallographic structure determination, allowing comparison of the model at various stages in the model building and refinement process, including the quick visual identification of subtle differences between non-crystallographic symmetry-related chains. Such information can be used to gain intuition regarding stability during refinement, the suitability of different refinement protocols and the degree of influence of any external restraints used. This can be useful in honing the refinement process, also allowing quick and easy identification of regions likely to be in drastic need of manual intervention.

### 5.4. Discussion   

The fact that crystallographically derived models have errors is often overlooked when performing structural analyses. It is important to remember that, whilst atomic coordinate data are static, macromolecules are actually dynamic in nature. Note that models are averaged over the range of conformations present in a heterogeneous crystal, which comprises a practically infinite ensemble of structures. This is reflected by positional uncertainty (parameterized as *B* factors) and, in the case of more extreme flexibility, missing atoms (disorder). Furthermore, model reliability may vary; some models may exhibit substantial incorrect regions, depending on data quality, crystallographic resolution and the presence of modeling errors. Consequently, it should be acknowledged that the usefulness and limitations of structural comparison are dependent on the quality of the compared models. Whilst we often assume a reasonable degree of experimental reliability and accuracy, the potential for model errors should not be overlooked. Indeed, some deposited models have been found to be incorrect (Bujnicki *et al.*, 2002 ▶; Chang, 2007 ▶), and even those that are considered correct cannot be considered perfect, as suggested by the improvements observed from the re-refinement of deposited models (Joosten *et al.*, 2009 ▶). There might be a temptation to account for model uncertainty when attempting to perform structural analyses, *e.g.* by weighting coordinates according to a measure of positional uncertainty. However, such an approach would fail to account for the correlated motion of close atoms, resulting in a measure of positional uncertainty relative to the coordinate frame of the crystal structure and not necessarily a measure of local conformational flexibility (as would be required for local analyses). With this in mind, it should be noted that model reliability should be considered (*e.g.* by inspection of the electron density) when performing structural analyses, remembering that the result of a structural comparison is simply a narrative, requiring a succinct contextual interpretation in order to be meaningful. It worth remembering that the static models under consideration are not flawless; experimentally derived models have errors and are in fact imperfect averaged snapshots of a dynamic structure. Whilst thermal parameters are often available (whether or not they are reliable), such a description is often a gross simplification of the actual system and does not capture information regarding the true conformational variability.

Because of the ever-increasing number of structures (and thus information) in the PDB available for exploitation, as time progresses there will be an increasing need for the provision of tools that allow easy navigation and extraction of relevant information. It seems reasonable that, at some point, the number of new structures or folds discovered will diminish, and the amount of truly unique structural information available will begin to saturate (Chothia, 1992 ▶). At such a point, the main challenge encountered by structural biologists may shift from experimental structure determination to navigation of data and extraction of information. This would heighten the necessity for effective and varied methods of comparative structural analysis. However, it would also require the ability to assess data quality so that subsequent interpretation is meaningful; whilst it is possible to infer information from data, the ability to gain knowledge is inherently limited by the validity of such information.

Structural comparison tools such as *ProSMART* can help break up the complexity that accompanies the constantly growing pool of structural data into a more readily accessible form, potentially offering biological insight, influencing subsequent experiments or injecting prior knowledge in order to aid structure determination. The development of complementary approaches for optimizing the usefulness of database resources, aiding the extraction of useful information, will undoubtedly become even more relevant in future.

## 6. The *LabDB* laboratory information management system   

### 6.1. Overview   

**Figure 5:**
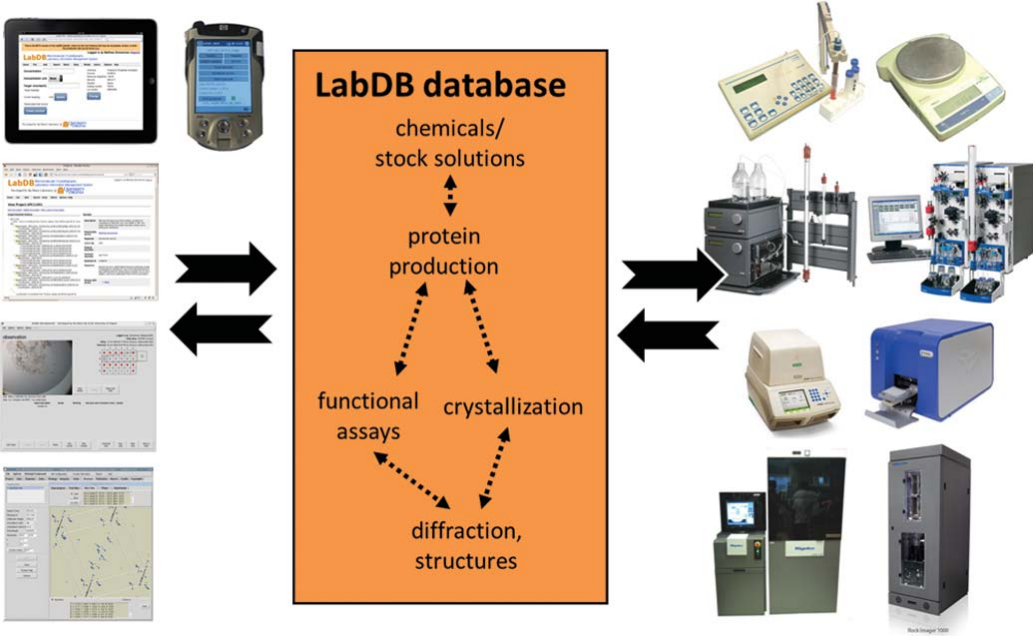
A graphical overview of the architecture of the *LabDB* LIMS. The different interfaces (the *LabDB* web GUI, *Xtaldb* and *HKL-3000*) are shown on the left, and examples of different laboratory instruments that connect to the system are shown on the right.

The *LabDB* laboratory information management system (LIMS) tracks, organizes and analyzes data for structure–function studies: chemical and solution management, protein production, crystallization, diffraction, structure solution, and *in vitro* biochemical and biophysical experiments. The system comprises multiple components specialized for different tasks (Fig. 5 ▶). Most of these components are accessed through a dynamic web-based interface (the *LabDB* GUI), though other stand-alone programs and modules also interact with the system, such as the *Xtaldb* system for crystallization, or the *hkldb* module of the *HKL-3000* suite (Minor *et al.*, 2006 ▶) for diffraction data collection and structure solution.

All of these components store the data they collect into a central *PostgreSQL* database, and thus all data collected by one component are made available to all the others. This is crucial given the highly interconnected nature of the different experiments in structure–function analysis. For example, the specific lot of a chemical used to prepare stock solutions for a crystallization experiment can have a major effect on whether crystallization is successful (McPherson, 1982 ▶). Similarly, details of the cloning construct and purification process [*e.g.* does the construct add an affinity tag and is that tag cleaved before analysis? (Majorek *et al.*, 2014 ▶)] can significantly alter the outcomes of ligand binding assays. In this way, *LabDB* provides a means of analyzing the experimental aspects of structure–function studies holistically and determining bottlenecks or other points of failure.

Whenever possible, the system collects data from laboratory hardware with minimal user intervention. Devices that may connect to or import data into *LabDB* include crystal observation robots, liquid handling robots, chromatography systems (GE Healthcare AKTA), quantitation tools (Caliper LabChip GXII and Bio-Rad Gel Doc EZ), reverse transcriptase polymerase chain reaction (RT-PCR) machines and isothermal titration calorimetry (ITC) systems (MicroCal iTC-200).

### 6.2. Modules of the *LabDB* system   

The *Reagents* component tracks laboratory chemicals, bottles and solutions. Detailed information is tracked about each chemical species, which is identified by SMILES representation (Weininger, 1988 ▶; Weininger *et al.*, 1989 ▶). Details of individual bottles of liquid or solid chemicals are stored, along with the manufacturer, quantity, date received *etc*. Details of stock solutions are stored, along with the name of the preparer and the date of preparation, and are linked back to the chemical bottle or ‘parent’ stock solution used to prepare them, such that all solutions in the laboratory have a full ‘audit trail’ back to the manufacturing lots of the reagents used to prepare them. All chemical bottles and stock solutions are also identified by unique barcode labels.

The *Reagents* component also integrates with hardware to simplify the process of creating stock solutions. The *LabDB* interface has been optimized to be used by mobile devices such as tablets or smartphones, and the system can be configured to connect with Mettler–Toledo balances and a variety of barcode scanners and label printers. When a researcher prepares a stock solution, she or he selects the concentration and volume desired, and scans the barcode of the reagent bottle using the mobile *LabDB* interface. The system calculates the amount of chemical to be added to reach the selected concentration, and the researcher weighs out the reagent on the balance until the calculated amount is approximately reached. The system then reads the true amount of reagent measured and adjusts the expected final volume of the solution accordingly. After the solution has been produced, a detailed and barcoded label is printed.

The *Protein Production* module tracks protein cloning, expression and purification. The system is optimized for recombinant expression of single proteins in prokaryotes, but is also capable of representing more complex types of data, such as cloning and expression of protein–protein complexes or purification of proteins from natural sources. Experimental data in *Protein Production* are represented in a hierarchical structure: one project contains one or more clones, which have one or more expressions *etc*. Each step also has detailed information about when and by whom a given experiment was performed. The system is equipped to handle data either from single experiments or in bulk, as multiple experiments can be imported from spreadsheet files.

The *Biochemical Assay* module tracks spectrophotometric binding and kinetics, thermal shift binding, ITC and protein quantitation. These tools associate functional and structural experiments, for example for selecting likely substrates for co-crystallization and soaking experiments. In addition to storing and displaying results for ‘single’ experiments (*e.g.* ITC binding curves or Michaelis–Menten plots), the tools for incorporating spectrophotometric and thermal shift results are designed to import results from 96- and 384-well plates. The thermal shift tool also parses raw data files from two types of real-time PCR systems: the Applied Biosystems 7900HT and the Bio-Rad C1000/CFX96 systems. The results are displayed graphically. For example, screening results from thermal shift assay plates are shown as a color-coded grid, where wells with greater shifts in melting temperature are shown in red and those with lesser shifts in blue.

The *Xtaldb* module is a stand-alone expert system for designing, tracking and analyzing the results of macromolecular crystallization experiments. *Xtaldb* allows for the design of either screening plates or custom optimization plates, using the sets of stock solutions prepared in the *Reagents* component. In the latter case the system also prepares pipetting instructions for the experimenter. The system records all observations of each drop, including images of the crystallization drops if available. The system also imports plate and screen designs and drop images from screening (Formulatrix Rock Maker and Emerald Opti-Matrix Maker) and observation (Rigaku Minstrel HT and Formulatrix Rock Imager) robotics.

In addition, *LabDB* is integrated with the *HKL-3000* suite for diffraction data collection and structure solution through the *hkldb* module, which provides access to all ‘upstream’ information about the reagents and protein purification. In *HKL-3000*, the diffraction and structure solution process can take advantage of this prior data, for example by identifying all compounds added to the protein preparation in the purification and crystallization process, to build a list of potential candidates for the identity of an area of unidentified density.

### 6.3. Reporting, analysis and future directions   

Two central objectives of the *LabDB* LIMS are real-time reporting of the status of the experimental pipeline and the ability to perform detailed analyses of the collected data. To this end the system provides extensive data-mining and analysis tools for translating raw experimental data into useful information. For example, there are a number of ‘dashboards’ with summary information, such as the number of experiments in each category by research or by project for a specified span of time. Each type of experiment is also fully searchable by most of the attributes of each. *Xtaldb* and *HKL-3000* also contain tools for preparing customized reports on various aspects of the data collected. New search tools and dynamic reports continue to be developed.

*LabDB* is used by two high-throughput PSI:Biology centers in the USA (MCSG and NYSGRC), as well as other major NIH consortia (the Center for Structural Genomics of Infectious Diseases and the Enzyme Function Initiative), and tracks millions of experiments on tens of thousands of targets. *LabDB* is still under active development, and future work includes the incorporation of additional types of experiment, support for data import from additional types of laboratory instrument, and a mechanism for tracking the locations of reagents and other materials by expanding the use of barcodes and near-field communication tags.

## 7. Data to knowledge: the Cambridge Structural Database   

### 7.1. Data   

The Cambridge Structural Database (CSD; Allen, 2002 ▶) contains crystal structures of organic molecules, some containing a metal. Since the first structures with coordinates in the 1930s (Robertson, 1936 ▶), this resource has grown to over 700 000 molecules. These individual structures can confirm the structural identity of a particular compound, perhaps its stereochemistry, how a metal atom is coordinated or even the identity of molecular species in a crystal structure, revealing hydrates, other solvates and cocrystals. We can also see the geometry of specific chemical groups and the conformation of particular molecules. Intramolecular interactions, for example hydrogen bonds, can be observed.

It was recognized at the outset that a collection of molecular structures would only be of limited value, and that knowledge bases capturing the geometry and interactions of the mol­ecules *as a collection* were of paramount importance. Indeed, it was with such thoughts in mind that the CSD was created. Speaking about herself and J. D. Bernal, Olga Kennard, who founded the CSD, recounted that, ‘We had a passionate belief that the collective use of data would lead to the discovery of new knowledge which transcends the results of individual experiments’ (Kennard, 1997 ▶).

Such information was originally published in hard-copy format, in the form of rather large books (Kennard *et al.*, 1971 ▶), but as the number of structures increased and technology allowed, electronic sharing took over.

### 7.2. Knowledge bases   

To allow access to derived data, the Cambridge Crystallographic Data Centre developed the CSD system, which includes the knowledge base *Mogul* (Bruno *et al.*, 2004 ▶). *Mogul* allows the user to retrieve population distributions corresponding to a wide range of structural groups. Such population distributions correlate well with calculated energy values (Allen, 2002 ▶) and are virtually instantaneous to retrieve. Unlike energy-based methods, which do not capture the behavior of all chemical groups equally well, data-led methods such as this are limited solely by the prevalence of appropriate systems in the database.

Although some supramolecular frameworks (for example metal–organic frameworks) have, to a first approximation, a single defined structure, some small organic molecules (for example drug-like molecules) have a range of accessible conformations. In such cases, the shape a molecule adopts is determined by a delicate balance between the conformational energy of the molecule, the energy it can gain from favorable interactions with neighboring molecules and the energy cost of any less-favorable interactions. Despite the fact that this must all be achieved against the backdrop of a limited range of symmetrical packing (Yao *et al.*, 2002 ▶), cases where the geometry of a molecule is significantly different from what one would expect are very rare. As such, the range of energy minima seen in a small-molecule crystal structure can be assumed to be representative of those seen in solution or when bound to a protein target.

The counterpart to this system in the area of molecular interactions is the knowledge base *IsoStar* (Bruno *et al.*, 1997 ▶). This system captures the geometry of non-bonded interactions between structural groups in molecules. Although the distributions of many interaction pairs are pre-calculated, a sister program, *Isogen*, allows one to generate population–geometry distributions for all interactions to be generated.

Our knowledge of molecular geometry and interactions is perhaps put to most direct use in the area of pharmaceutical and agrochemical design. Numerous examples exist that refer to the optimization of molecular geometry, particularly with respect to the manipulation of torsion angles with the intent of increasing the binding potency of a molecule to its target (Brameld *et al.*, 2008 ▶), as do many for the optimization of interactions (Bissantz *et al.*, 2010 ▶).

### 7.3. Application of knowledge in software   

The direct use of knowledge extracted from small-molecule structures is evident, but much use goes relatively unnoticed (Taylor, 2002 ▶). For example, in addition to use in small-molecule crystallography, the restraints used in the refinement of both ligand and protein structures are often derived from small-molecule crystal structures (Engh & Huber, 1991 ▶). Furthermore, when exploiting these structures, for example through protein–ligand docking, small-molecule structures play a key role in the parameterization of many scoring functions (Velec *et al.*, 2005 ▶).

As databases such as the CSD continue to grow, so does the knowledge we are able to extract from them. We are now at the stage where knowledge extracted from existing crystal structures is used in the assignment of chemical functionality to coordinates from a structure determination (Macrae *et al.*, 2008 ▶). Statistical studies of the enrichment of specific interactions in crystal structures enable us to understand just which interactions drive molecular associations (Taylor, 2014 ▶) and, combined with our understanding of molecular conformations, this is bringing us closer to the point whereby we can predict the very crystal structures themselves (Bardwell *et al.*, 2011 ▶; Kazantsev *et al.*, 2011 ▶).

## 8. Conclusions   

Like other modern areas of science, structural biology faces enormous challenges created by the vast amount of data generated every day by research groups. Only rarely are raw data exported from the research laboratory. Rather, the results of data analysis (information) are published, in the form of research papers, and depositions of models and reduced data in various repositories. The deposition of these models and reduced data is often required by the journals and/or funding agencies, but is sometimes treated as a nuisance by researchers. For this reason the contents of different repositories are not always consistent with one another, and sometimes are not even self-consistent in themselves, making the analysis of data in aggregate very difficult.

In many cases, there are no suitable repositories or databases for raw data at all. For example, while the models and processed structure factors produced in macromolecular X-ray crystallography experiments may be submitted to the PDB, there is no corresponding repository for the diffraction images, even though these images comprise the primary data collected. The advantages of preserving such data are numerous: they provide the ability to verify models, to assess data quality better and to produce better models in the future when methodological improvements are made. All of these advantages make it possible to improve systematically the structural data contained within the PDB, which would in turn aid further structure determination and bioinformatics work.

The ripple effect of suboptimal information is frequently underestimated by individual depositors and very difficult to measure. Quite often, the software necessary to extract useful information is complicated, difficult to use and more costly than the instruments that generate data. There is hope that the implementation of ‘Big Data’ tools may partly cure the present situation. However, tools, techniques and technologies that effectively support data harvesting, data mining, computations and the sharing of data with collaborators (*i.e.* that make data available in a straightforward way) are very difficult to develop and require a much greater investment than simply assembling massive computational clusters with petabyte cloud storage. The creation of a smooth path from data to knowledge will require a group of talented individuals, to­gether with creativity and long-term vision on the part of their leaders. Last but not least, these groups will need significant resources to develop tools that effectively address issues related to the non-reproducibility of experimental results and to implement the systems necessary to pave the data-to-knowledge road.
